# The Role of Radiotherapy in the Management of Vaginal Melanoma: A Literature Review with a Focus on the Potential Synergistic Role of Immunotherapy

**DOI:** 10.3390/jpm13071142

**Published:** 2023-07-16

**Authors:** Francesco Cuccia, Salvatore D’Alessandro, Livio Blasi, Vito Chiantera, Giuseppe Ferrera

**Affiliations:** 1 Radiotherapy Unit, Radiation Oncology, ARNAS Civico Hospital, 90100 Palermo, Italy; titodales@gmail.com (S.D.); giuseppe.ferrera@arnascivico.it (G.F.); 2 Radiation Oncology School, University of Palermo, 90100 Palermo, Italy; 3 Medical Oncology, ARNAS Civico Hospital, 90100 Palermo, Italy; livio.blasi@arnascivico.it; 4 Gynecological Oncology, ARNAS Civico Hospital, 90100 Palermo, Italy; vito.chiantera@arnascivico.it

**Keywords:** vaginal melanoma, radiotherapy, immunotherapy

## Abstract

Among the mucosal melanomas, vaginal melanomas are very rare tumors, accounting for less than 20% of melanomas arising from the female genital tract. They occur most frequently in women in post-menopausal age, but younger patients may also experience this neoplasm, mainly located in the lower third of the vagina or the anterior wall. The optimal management of this tumor remains controversial, with surgery reported as the most frequently adopted approach. However, a clear benefit of surgical treatment in terms of survival has not yet been demonstrated. Conversely, radiotherapy may represent an attractive non-invasive alternative, and there are several favorable reports of the role of radiation therapy, either delivered with photons, brachytherapy, or hadrontherapy. A wide range of techniques and fractionation regimens are reported with substantially good tolerance to the treatment, and acute G3 or higher toxicities are reported only in the case of concurrent immunotherapy. Of note, due to the rarity of the disease, there is a lack of high-level evidence for the optimal therapeutic option. In this scenario, recent studies theorize the possibility of developing combinatorial approaches of radiotherapy with immunotherapy based on cutaneous melanomas reports. In this review, we aim to summarize the evidence available in the literature supporting the role of definitive radiotherapy for vaginal melanomas, with a focus on the combination of RT with immunotherapy, in terms of optimal timing and biological rationale.

## 1. Introduction

Vaginal melanomas (VM) are a rare entity among the mucosal melanomas, accounting for less than 20% of melanomas located in the female genital tract. Clinical staging of VM is derived from cutaneous disease, and tumor size and lymph-nodal metastases are related to a poorer prognosis, with an overall survival rate of 5 years ranging between 5% and 25% [1].

It more commonly affects women in the post-menopausal period between 60 and 70 years of age, but sometimes it might also be observed in younger patients. The preferred site of presentation is usually the lower third of the vagina or the anterior vaginal wall. Melanocytic presentation is the most common occurrence, although very rare cases of non-melanocytic diseases are reported in the literature. Disease staging is based on the International Federation of Gynecology and Obstetrics(FIGO) classification for vaginal cancer, although TNM classification is also widely adopted, and the depth of invasion is considered an important risk factor, such as for cutaneous melanomas. 

Due to the rarity of the disease, optimal management remains a matter of debate. Unlike non-melanoma vaginal cancer, where surgery has a limited role in very early stages or advanced stage IV disease with healthy structures invasion, for vaginal melanomas, surgical resection is the preferred approach, although with a debated survival benefit and a wide spectrum of complications, such as infection, bladder or urethral damage, fistulas, or hemorrhages [2].

Furthermore, diagnosis occurs more frequently in the advanced stages of the disease, with a reduced probability of surgical radicality and a higher risk of severe sequelae. However, combined approaches with adjuvant radiotherapy have resulted in very poor outcomes due to the high rates of local and distant recurrences [3].

Systemic therapy options for mucosal melanoma are limited, given the reduced response to conventional cytotoxic drugs. Also, BRAF mutations are rarely present in mucosal melanomas, compared to cutaneous disease, while, KIT mutations are more frequently reported [4].

In this scenario, immunotherapy has recently been introduced with a sensitive improvement in clinical outcomes, reporting objective response rates ranging between 20% and 75%, with durable responses reported in the literature [5].

Although response rates to immunotherapy are lower for mucosal melanomas compared to cutaneous melanomas, the possibility of combining radiotherapy with immunotherapy is an attractive treatment option in this setting [6,7].

Melanoma is traditionally considered a radio-resistant tumor with a natural capacity to repair damages caused by low-dose radiotherapy. These features of the disease have led clinicians to explore alternatives to conventional external beam radiotherapy (EBRT), such as brachytherapy or hadrontherapy, both of which can deliver higher doses to the target with limited exposure to organs at risk. 

In recent years, based on the radiobiological characteristics of VM, the use of stereotactic body radiotherapy (SBRT) has also been increasingly reported, not only as a therapeutic option able to overcome the intrinsic radio-resistance of melanoma but also for its potential effect on enhanced immunogenicity [8].

SBRT allows clinicians to deliver very high doses to small volumes, with a steep dose gradient and a reduced low-dose bath of healthy nearby structures [9].

Some studies have favorably reported the combination of high-dose radiotherapy with immunotherapy, with improved clinical outcomes in comparison with patients treated with immunotherapy alone [10,11,12,13].

As surgery remains the preferred treatment option for the management of VM, radiotherapy may represent an effective alternative for patients unfit for surgical excision, with a further potential benefit provided by immunotherapy. 

In this literature review, we aim to collect the available evidence in support of the use of radiotherapy for VM, with a focus on the potential synergistic use of immunotherapy. 

## 2. Methods

A review of the literature was performed in May 2023 to select articles reporting on the safety and efficacy of definitive radiotherapy for vaginal melanoma. Publications reporting the synergistic use of radiotherapy and immunotherapy were also considered for the purpose of this study. Adjuvant or neoadjuvant radiotherapy cases were not considered for the purpose of this study. We searched the PubMed, Scopus, and Embase databases to retrieve papers containing combinations (by AND/OR) of the following search terms: “vagina”, “melanoma”, “radiotherapy”, “stereotactic radiotherapy”, and “radiation therapy”. Only the articles published between January 1995 and April 2023 were considered eligible for the present study. All the studies selected as potentially eligible for this review were screened by two authors (FC and SDA), with a third author (GF) involved in the case of disagreement between the first two. 

Due to the rarity of the disease, case reports were considered eligible for the purpose of the present study. Non-English publications, reviews, and studies involving patients treated with surgery were excluded. 

The following data were extracted from all the selected publications: first author and year of publication, number of patients treated, median age, study design, intent and radiotherapy fractionation, radiotherapy technique, any concurrent systemic therapies, clinical outcomes (local control, LC; overall survival, OS), and grades and incidence rates of adverse events. Median follow-up was calculated starting from the end of radiotherapy; LC was defined as the interval between the end of treatment and the last follow-up known or the occurrence of a disease relapse within the radiotherapy field; OS was defined as the interval between the end of treatment and the last follow-up known or the occurrence of death by any cause. 

## 3. Results

Eighty-five studies were initially identified from the literature research. Based on the abovementioned criteria of inclusion/exclusion, a total of 11 studies were considered relevant for the present literature review. Seven studies were case reports and four were reports on the outcomes of retrospective series; no prospective studies were found [7,14,15,16,17,18,19,20,21,22,23] (Table 1).

The synergistic use of immunotherapy is reported in six publications [6,16,17,19,20,21]. A total of 36 patients were treated with definitive radiotherapy for vaginal melanoma, consisting of conventional external beam radiotherapy in 5 cases, stereotactic radiotherapy in 5 cases, hadrontherapy in 25 cases, and brachytherapy in 1 case. The median patient age was 70 years (range, 55–80 years) and tumor size ranged between 7 mm and 80 mm. 

### 3.1. Imaging Modalities and Diagnostic Process

Physical examination and disease biopsy were performed in all the cases treated. The preferred imaging modality for local staging was contrast-enhanced pelvic magnetic resonance imaging (MRI) in all studies, whereas positron emission tomography (PET) with fluorodeoxyglucose was performed in seven studies for local and global staging. 

### 3.2. Radiotherapy Techniques 

A wide range of radiotherapy techniques and treatment schedules is reported, with hadrontherapy being the most frequently applied approach, followed by EBRT delivered with either 3D conformal radiotherapy or intensity-modulated radiotherapy. Conventional doses ranged between 45 Gy and 70 Gy using 2 Gy per fraction regimens; stereotactic radiotherapy was reported in five patients with doses ranging between 24 Gy and 30 Gy in 3–5 fractions. Treatment was generally delivered on consecutive days, and several techniques are reported. An exception is reported in the study by Parisi et al., in which SBRT was delivered once weekly on days 0, 7, and 21 [20]. 

Brachytherapy (BRT) is reported in a single study, with ^103^Palladium BRT implant as a boost after conventional EBRT. 

Hadrontherapy with carbon ion RT is reported in three studies for a total of 25 patients, with a median dose of 38.7 Gy_RBE_ plus a boost of up to 63 Gy in a median number of 16 fractions. 

### 3.3. Combination with Immunotherapy or Other Systemic Treatments

Combination with immunotherapy is reported in six studies, including a case report of a metastatic patient treated at all the disease sites with dual immunocheckpoint inhibitors (nivolumab + ipilimumab), followed by maintenance nivolumab. In the remaining cases, immunotherapy was initiated concurrently with radiation therapy and maintained until disease progression. In all the cases reported, immunotherapy was combined with stereotactic radiotherapy, except for two patients treated with conventional EBRT, including one receiving a sequential brachytherapy boost. 

Conventional chemotherapy was administered in two cases, including dacarbazine in one study, and temozolomide combined with tyrosine kinase inhibitors in another study. 

One patient concurrently received interferon 2-α.

### 3.4. Clinical Outcomes

With a median follow-up of 15 months, local recurrence was observed in two cases, with patients who were candidates for surgery or systemic treatment as a second-line approach. The study by Murata et al. [18] reported 2-year LC rates of 71%. The median distant progression was 6 months (range, 2–12 months) with six patients who died of progressive disease. 

Globally, radiotherapy is well tolerated, with a low incidence of G3 or higher adverse events. Six cases of acute G3 toxicity (mainly skin toxicity) were reported in patients with concurrent immunotherapy (three patients with ipilimumab, one with nivolumab, and two with interferon). Two patients received SBRT, three were treated with CIRT, and one with conventional EBRT. One patient developed acute G3 colitis when SBRT (30 Gy/5 fractions) was combined with concurrent ipilimumab. 

## 4. Discussion

The optimal management of VM remains a matter of investigation due to the rarity of the disease. The literature reports surgery as the most frequently adopted approach. However, the extent of surgical resection may vary from wide excision to pelvic exenteration, including a more aggressive approach to the lymph nodes when compared to vulvar disease. Moreover, a clear benefit in terms of survival has not yet been demonstrated [24].

In this scenario, modern radiotherapy techniques have emerged as a potential non-invasive alternative. 

Initial experiences of brachytherapy alone or in combination with EBRT are reported in the literature, suggesting the feasibility of delivering high doses to the tumor using BRT, especially as a boost after conventional EBRT [16].

Hadrontherapy has recently been reported as a potential therapeutic option for mucosal melanomas due to its higher radiobiological effect, useful in the case of radio-resistant tumor histologies, and for the peculiar dose distribution to optimally spare nearby healthy structures [25].

In addition to the major sparing of the proximal organs at risk, the higher release of energy in a single point, compared to photons, is supposed to result in a higher probability of double-strand DNA breaks, with a consequent enhanced effect of tumor cell killing [26].

All these features justify the potential of hadrontherapy for radio-resistant tumors, although the global availability of this technology is quite limited to date. 

Murata et al. [18] reported the outcomes of one of the largest series of female genital tract melanomas, including 22 patients with VM, treated with CIRT. Despite referring to a series of patients with mixed primary sites, the authors reported excellent rates of complete response (about 81%) with 2-year LC, OS, and DPFS rates of 71%, 53%, and 29%, respectively. Interestingly, only three acute grade 3 skin adverse events were recorded. 

Similar outcomes are reported in a smaller series by Barcellini et al. [19] including two patients with VM treated with CIRT and by Ohno et al. in a single case report [15].

Beyond the role of hadrontherapy, another topic of potential interest for the treatment of VM relies on the combination of radiotherapy with novel systemic agents. 

For this purpose, an interesting case is reported by Yin et al. [23] of a 55-year-old patient with metastatic VM, who received conventional EBRT in combination with temozolomide + tyrosine kinase inhibitors, with good results in terms of local control but short-term distant progression.

In recent years, the combination of radiotherapy with immunotherapy has attracted increasing interest, as reported in other clinical settings, especially for non-small cell lung cancer, kidney cancer, cutaneous melanomas, and cutaneous squamous cell carcinoma [27,28].

Starting from pre-clinical studies that suggest a major immunogenic effect from radiotherapy when delivered using higher doses per fraction (i.e., 8 to 10 Gy for 1–3 fractions) [29], several studies highlight SBRT as a means of releasing neo-antigens capable of activating and proliferating T-cells against tumor cells. This effect favorably combines with the intrinsic activity of recruiting T-cells mediated by immunotherapy [28]. Moreover, SBRT, compared to conventionally fractionated radiotherapy, generates higher endothelial vascular damage, which facilitates the delivery of targeted therapies to the tumor [30].

Several reports in the literature, specifically for cutaneous melanoma, support the combination of immunotherapy with high-dose SBRT, particularly for brain metastases [31,32].

On the contrary, the combination of SBRT with immunotherapy for extracranial melanoma is currently a matter of debate, with some studies reporting a detrimental impact of radiotherapy when combined with immunotherapy [33,34].

In contrast, Youland et al. [35] reported improved outcomes for extracranial melanomas when SBRT is chosen as the preferred approach, with a statistically significant impact on local control, distant progression, and overall survival in a cohort of 75 patients.

This conflicting evidence highlights the need to further investigate the optimal combination of radiotherapy with immunotherapy, not only in terms of dose and fractionation but also (and probably with more interest) for the sequencing of these two therapeutic tools [36].

A majority of the literature supports the beneficial effect of upfront SBRT followed by immunotherapy in order to enhance the response of the immune system, and other combinations are also reported in the scientific community. Fenioux et al. recently reported favorable outcomes in a cohort of 62 patients with brain metastases from melanoma when immunotherapy was delivered for a long interval (12 weeks) prior to stereotactic radiosurgery [36].

A recent literature review published by Tian et al. reported improved outcomes for brain metastases when concurrent radiotherapy and immunotherapy were delivered in terms of clinical outcomes, but at the same time, the authors focused on the need for a precise definition of “concurrent administration” in terms of time intervals. Starting from an analysis of the biological mechanisms of both SBRT and immunotherapy, the authors hypothesized a peak in the immunogenic effect of high-dose radiotherapy within 24–72 h from the end of treatment, suggesting that right after this interval, immunotherapy has a higher benefit due to the radiotherapy boost effect [37].

Of note, a major incidence of adverse events is reported in the case of concurrent SBRT with immunocheckpoint inhibitors. 

Despite being referred to as cutaneous melanoma, all these data suggest the need for better design prospective studies with precise time intervals between SBRT and immunotherapy since this feature might be crucial for an optimal combinatorial response.

Nonetheless, another caveat in the combination of radiotherapy (either with conventional fractionation or SBRT) with immunotherapy is represented by the potential increased risk of severe adverse events [38].

Mesko et al. [17] reported a case of a 70-year-old patient who received conventional EBRT (45 Gy + electron boost up to 63 Gy) for a VM with concurrent ipilimumab. Despite reporting severe acute skin toxicity, the patient remained free of disease after 15 months, with a complete response on PET-FDG scan. Data on skin toxicity with concurrent radiotherapy and ipilimumab are limited, but the authors addressed this phenomenon to the high dose of the target and the size of the planning target volume (PTV). 

Schiavone et al. [7] reported the outcomes of a case series including three patients with VM treated with concurrent radiotherapy and immunotherapy, with two patients treated with 30 Gy/5 fx with good tolerance. Notably, two patients in this series also received surgery after the radiotherapy treatment in order to maximize local control. 

In light of the higher risk of toxicity when administered concurrently with immunocheckpoint inhibitors, SBRT appears to be a more attractive alternative, as it is usually delivered in smaller volumes compared to the larger volumes of conventional EBRT, and SBRT also offers the abovementioned potential to enhance the response to immunotherapy. 

In this scenario, favorable responses are reported. Parisi et al. [20] reported a case of complete remission of VM treated with volumetric modulated arc technique (VMAT)-based SBRT (24 Gy/3 fx once a week) plus pembrolizumab in an 80-year-old patient unfit for surgical approaches. This report highlights the feasibility of stereotactic approaches in older cancer patients, suggesting the potential synergistic role of radiation therapy and immunotherapy, especially when radiotherapy is delivered at higher doses per fraction [27]. 

In agreement with this, Schonewolf et al. [22] reported a similar case in an 80-year-old patient unfit for surgery treated with SBRT (30 Gy/5 fx) combined with pembrolizumab, obtaining a complete metabolic response not only to the site of the primary tumor but also to locoregional metastases (not included in the RT field), suggesting a potential abscopal effect. In the same study, the authors also reported similar good outcomes in another case (not included in the present review) of a 56-year-old patient who received SBRT plus immunotherapy and subsequent surgery on the primary tumor.

Sezen et al. [21] reported the outcomes of a 73-year-old patient with upfront metastatic VM who received concurrent nivolumab and ipilimumab with palliative 30 Gy/5 fx to the primary tumor site for vaginal bleeding. The subsequent PET-FDG scan detected a complete response to the site of SBRT, and the onset of three liver metastases and one to the right groin. All these sites received SBRT treatment in combination with double immunotherapy followed by nivolumab monotherapy maintenance, with the patient reaching a complete metabolic response 32 months after the diagnosis. This study reinforces the hypothesis of high-dose radiotherapy as a sort of in-situ vaccine, able to facilitate the release of antigens enhancing the immune response to tumor cells [28,39].

Furthermore, it is also theorized that low-dose radiotherapy may have a favorable combination with immunotherapy by increasing the T-cell homing, thus evoking a systemic response with a different biological mechanism than the well-known abscopal effect [40,41].

The present literature review has some limitations. First of all, the available evidence mostly consists of case reports or retrospective series with very limited follow-ups and different doses and techniques. In order to assess the sole impact of radiotherapy in the management of VM, only curative treatments were considered for the purpose of this analysis. 

We also proposed a focus on a favorable combination with immunotherapy, aiming to translate the knowledge derived from cutaneous melanoma. 

Also, this field appears to have several areas to be fully explored, starting from the exact interaction between radiotherapy and immunotherapy in terms of biological mechanisms to the need to precisely identify the optimal sequencing of these two therapeutic tools [42].

In conclusion, the optimal treatment for vaginal melanoma remains a matter of debate, with surgery, radiotherapy, and immunotherapy as the three main strategies currently available. Of course, as surgery remains the most frequently preferred treatment option, radiotherapy could be considered for patients unfit for invasive approaches. In this scenario, this literature review reports the substantial feasibility of exclusive radiotherapy with or without novel systemic agents. Studies with larger sample sizes are advocated, but in light of the currently available limited evidence, multidisciplinary management of patients with VM is considered mandatory. 

## Figures and Tables

**Table 1:** Reports of definitive radiotherapy for vaginal melanomas.

Author (Year)	N. of Patients Treated	Age	Radiotherapy Setting	RT Technique	Radiotherapy Schedule	Concurrent or Sequential Systemic Treatment	G ≥ 3 Toxicity	Clinical Outcomes
Petru et al. (1998) [14]	2	73	definitive	EBRT	50 Gy/25 fx	none	none	1 local recurrence after 20 months, 1 distant progression after 2 months
Ohno et al. (2007) [15]	1	55	definitive	CIRT	57.6 Gy_RBE_/16 fx	dacarbazine	none	Complete response after 12 months, death by disease after 18 months
McGuire et al. (2008) [16]	1	71	definitive	EBRT + BRT	46 Gy/23 fx + BRT ^103^Pd implant 100 Gy	none	none	After 12 months local control, distant progression due to polymetastatic spread
Schiavone et al. (2016) [7]	3	61.5	definitive/neoadjuvant (2 patients received surgery about one month after RT)	EBRT (SBRT)	30 Gy/5 fx (*n* = 2); 60.2 Gy/28 fx (*n* = 1)	ipilimumab	1 acute G3 colitis; 1 acute G3 skin toxicity	2 patients NED; 1 death by disease after 16 months
Mesko et al. (2017) [18]	1	70	definitive	EBRT (photons + electrons)	45 Gy/25 fx + electron boost up to 63 Gy	ipilimumab	1 acute G3 skin toxicity	NED after 15 months of follow-up
Murata et al. (2019) [19]	22	71	definitive	CIRT	36 Gy_RBE_ + boost up to 64 Gy_RBE_/16 fx	Interferon (*n* = 9); nivolumab (*n* = 1)	3 acute G3 skin toxicities	2 years LC = 71%; 2 years OS = 53%; 2 years DPFS = 29%
Barcellini et al. (2019) [20]	2	60.5	definitive	CIRT	38.7 Gy_RBE_/9 fx + boost up to 68.8 Gy_RBE_	none	none	1 patient developed distant progression after 12.6 months; 1 patient received salvage surgery after 11 months for suspected local recurrence
Parisi et al. (2020) [20]	1	80	definitive	EBRT (SBRT)	24 Gy/3 fx	pembrolizumab	none	Complete response after 12 months; NED at 18 months of follow-up
Sezen et al. (2021) [21]	1	73	palliative (metastatic patient)	EBRT (SBRT)	30 Gy/5 fx	ipilimumab + nivolumab	none	NED after 32 months from initial diagnosis (patients treated to 4 metastatic sites with SBRT in combination with immunotherapy)
Schonewolf et al. (2021) [22]	1	80	definitive	EBRT (SBRT)	30 Gy/5 fx	pembrolizumab	none	Complete local response + potential abscopal effect
Yin et al. (2022) [23]	1	55	definitive	EBRT	70 Gy/35 fx	temozolomide + anlotinib + toripalimab	none	24 months local control, distant progression after 6 months

Abbreviations: CIRT = carbon ion radiotherapy; DPFS = distant progression-free survival; EBRT = external beam radiotherapy; LC = local control; NED = no evidence of disease; OS = overall survival; RBE = relative biological effectiveness.

## Data Availability

Data available on request.
